# miR-133b, a particular member of myomiRs, coming into playing its unique pathological role in human cancer

**DOI:** 10.18632/oncotarget.16745

**Published:** 2017-03-31

**Authors:** Daojiang Li, Lu Xia, Miao Chen, Changwei Lin, Hao Wu, Yi Zhang, Songqing Pan, Xiaorong Li

**Affiliations:** ^1^ Department of General surgery, The Third Xiangya Hospital of Central South University, Changsha, Hunan 410013, China; ^2^ Center for Experimental Medicine, The Third Xiangya Hospital of Central South University, Changsha, Hunan 410013, China; ^3^ Department of Neurology, Renmin Hospital of Wuhan University, Wuhan 430060, China

**Keywords:** cancer, miR-133b, tumor, myomiRs, microRNAs

## Abstract

MicroRNAs, a family of single-stranded and non-coding RNAs, play a crucial role in regulating gene expression at posttranscriptional level, by which it can mediate various types of physiological and pathological process in normal developmental progress and human disease, including cancer. The microRNA-133b originally defined as canonical muscle-specific microRNAs considering their function to the development and health of mammalian skeletal and cardiac muscles, but new findings coming from our group and others revealed that miR-133b have frequently abnormal expression in various kinds of human cancer and its complex complicated regulatory networks affects the tumorigenicity and development of malignant tumors. Very few existing reviews on miR-133b, until now, are principally about its role in homologous cluster (miR-1, −133 and -206s), however, most of constantly emerging new researches now are focused mainly on one of them, so In this article, to highlight the unique pathological role of miR-133b playing in tumor, we conduct a review to summarize the current understanding about one of the muscle-specific microRNAs, namely miR-133b, acting in human cancer. The review focused on the following four aspects: the overview of miR-133b, the target genes of miR-133b involved in human cancer, the expression of miR-133b and regulatory mechanisms leading to abnormal expression of miR-133b.

## INTRODUCTION

MicroRNAs (miRs) are endogenous short non-coding RNA molecules (containing about 22 nucleotides) found in various species, which functions in degradation and translational inhibition by perfect of imperfect complementarities with specific sequences of mRNA [1, 2], in this manner a single miR can regulate a large amount of independent targeted gene and involve in a variety of physiological and biochemistry process including diverse cellular signaling and metabolic pathways, as well as basic cell proliferation, differentiation, apoptosis. In addition, abnormal miRs expression is a hallmark of several diseases, including cancer [3, 4]. Some miRs, *per se, have* a substantial influence on disease progresses and been extensively studied and characterized, such as the canonical myomiR-133b. miR-133b belong to canonical muscle-specific microRNAs (myomiR) families(miR-1, −133 and -206s) and was initially considered muscle specific in that it was highly enriched in heart and skeletal muscle and played a critical regulator for muscle development and remodeling [5, 6]. But now more and more researches coming from our group and others indicated that miR-133b presented aberrant expression in various kinds of human cancer and might be closely associated with the occurrence and development of tumor.

Until now, there are 3 available reviews about miR-133b in English bibliographic databases. they are “Roles of the canonical myomiRs miR-1, -133 and -206 in cell development and disease” [6], “microRNA-133: expression, function and therapeutic potential in muscle diseases and cancer” [7] and “microRNA-1/133a and microRNA-206/133b clusters:Dysregulation and functional roles in human cancers” [5], respectively. However, discussions from these excellent reviews are mainly centered on the role of miR-133b in homologous cluster (miR-1, -133 and -206s) and sometimes its unique physiological functions are harder to distinguish and to be highlighted extremely well. Furthermore, most of researches springing up now in our group and others are focused mainly on one of myomiR and simultaneously found different myomiR isomer genes to be independently expressed under cell specific regulation, even discrepancy can occur in single myomiR. Therefore, in order to avoid ambiguous identification and highlight the unique pathological properties of miR-133b playing in human cancer, we made a review only for miR-133b. Firstly, we introduced the general situation of miR-133b, and then summarized the current understanding about functional significance and the aberrant expression of miR-133b acting in human cancer.

### The overview of miR-133b

**Figure 1:**
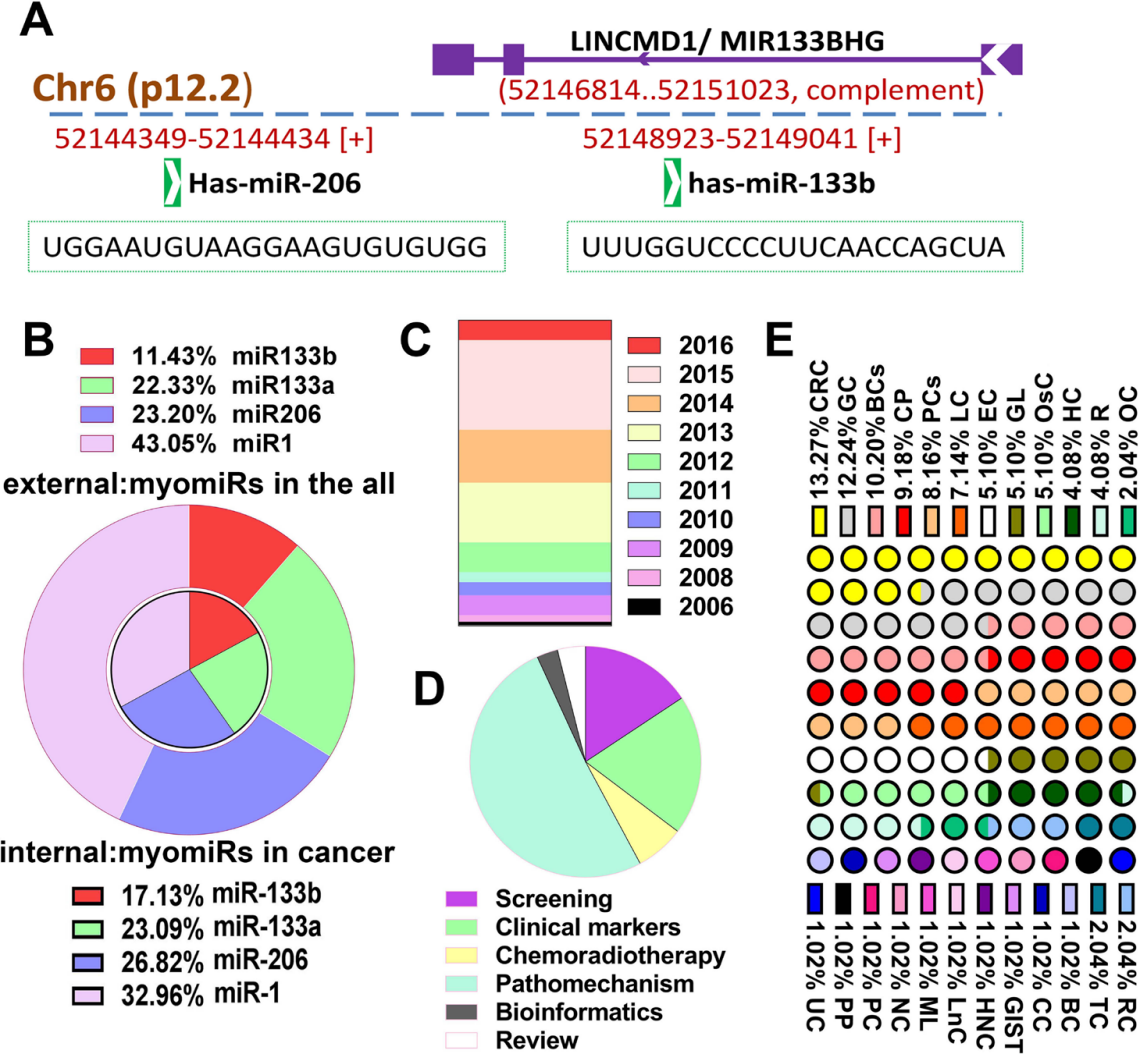
The overview of miR-133b (**A**) The representative gene structure of miR-133b/miR-206 clusters and the mature miRNA sequence of these two miRNAs; miR-133b/miR-206 clusters located in on chromosomes 6p12.2 and miR-133b gene transcript is located within the precursor of the long non-coding RNA linc-MD1. (**B**) The research proportion of 4 different myomiRs in all literatures and the literatures on cancer. The study of miR-133b is proportionately less and more research of it is about caner. (**C**) The year distribution of study of miR-133b with cancer. (**D**) The research categories of miR-133b were involved in tumor, the majority of the study about miR-133b focuses on pathomechanism. (**E**) The proportion of miR-133b in various cancers, the majority of the study about miR-133b focuses on digestive tract neoplasms. Bladder cancer (BCs); colorectal cancer(CRC); hepatocellular carcinoma(HC); lung cancer(LC); glioma(GL); ovarian cancer(OC); prostate cancer(PCs); gastric cancer(GC); pancreatic cancer(PC); esophagus cancer(EC); myeloid leukemia(ML); osteosarcoma(OsC); renal carcinoma(RC); breast cancer(BC); pheochromocytoma and paraganglioma(PP); Uterine sarcoma(UC); small bowel gastrointestinal stromal tumors (GIST); review(R); cervical carcinoma(CC); head-neck carcinoma(HNC);Carcinoma of Tongue(TC); laryngeal cancer(LnC); nasopharyngeal carcinoma(NC); comprehensive papers involved several human cancer(CP).

MicroRNA families miR-1 and miR-133, and single miR-206 are collectively known as the muscle-specific miRNAs (“myomiRs”) because of they are highly conserved in the musculatures across species [8]. miR-133b and miR-206, two isomers of miR-1 and miR-133a form different clusters located in on chromosomes 6p12.2, 20q13.33 and 18q11.2, respectively. Although these miRs have the same specificity of tissue expression, mature miRNA sequence present variant nucleotiedes (Figure 1A shows the representative gene structure of miR-133b/miR-206 clusters and each mature miRNA sequence), this also explains to a certain extent its function may be different. Recently, burgeoning literatures have found that these gene clusters also play an import role in human cancer apart from muscle tissue. To avoid the repetition and encumbrance of related contents, and help focus on unique pathological role of miR-133b in cancer, more minute details about myomiRs can be found in these excellent review papers [5, 6, 9]. For a more comprehensive understanding the latest updates and condition of miR-133b properties in cancer regulatory networks and tumor biology, Firstly, we performed an extremely simple mathematical analysis. Literature searches (Pubmed) in the Figure 1B have showed the research proportion of 4 different myomiRs in all literatures and in article on cancer, year distribution of miR-133b with cancer was presented in Figure 1C, which indicated the study of miR-133b is proportionately less and growing annually, remarkably, it seem that more research of miR-133b is about caner. At the same time, we also analyzed the proportion of miR-133b in various cancers, and research categories of miR-133b involved in tumor, the result indicated that the majority of the study about miR-133b focuses on pathomechanism and digestive tract neoplasms (Figure 1D and 1E). In addition, another original intention for this review is that there has been individual review for each myomiRs but miR-133b, these independent reviews about miR-133 [7]/a [10], miR-1 [11–13], miR-206 [14, 15] and miR-133b which we review in here is so particularly important for us to understand the jointly or independently role of myomiRs acting in human disease.

### The target genes of miR-133b involved in human cancer

**Figure 2:**
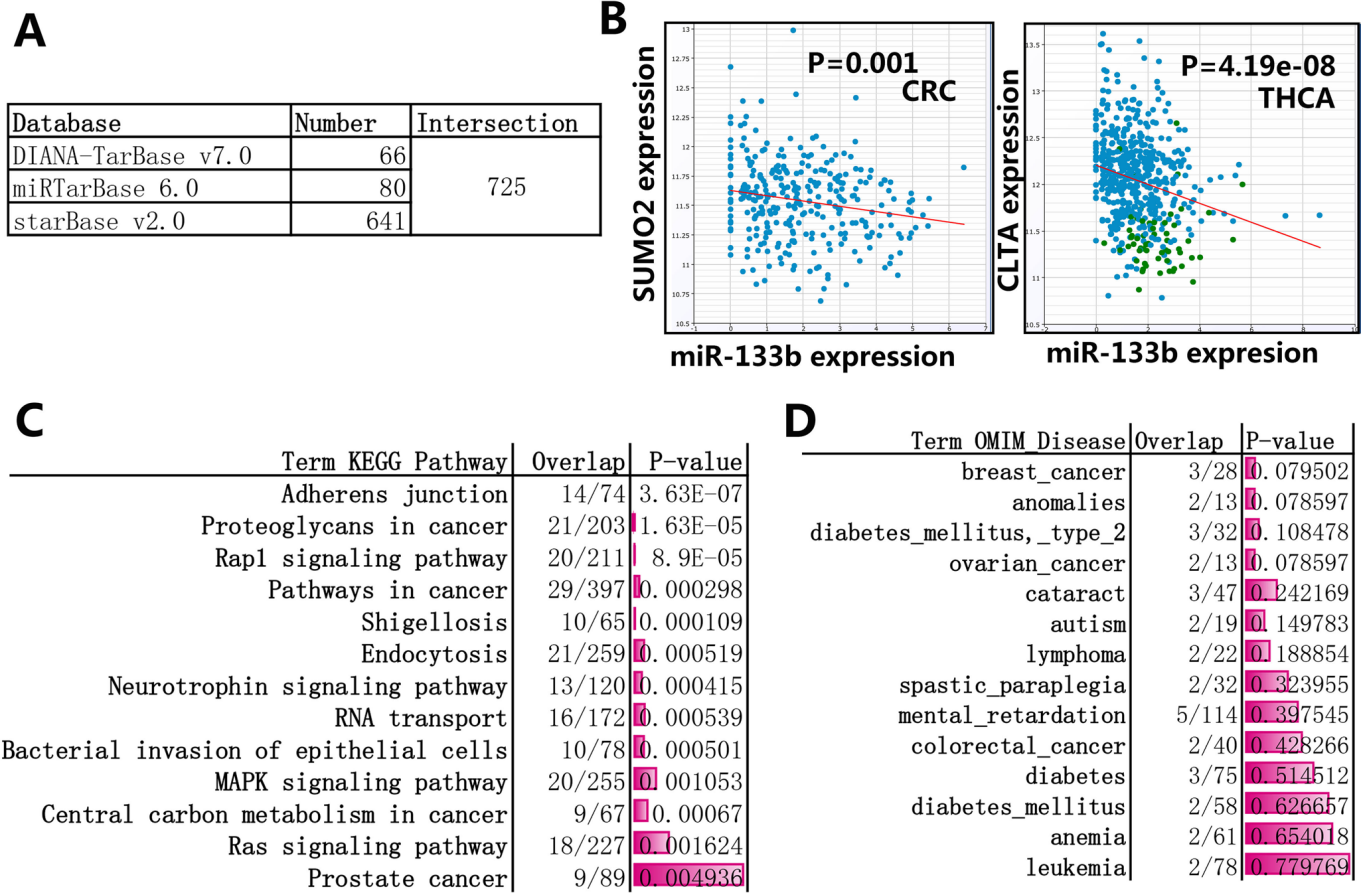
Enrichment analysis of the target genes of miR-133b (**A**) the experimentally validated genes and predicted targets supported by CLIP-Seq data were selected, the database miRTarBase 6.0 and DIANA-TarBase v7.0 provieded 80 and 66 experimentally validated genes, respectively; the starBase v2.0 provided 641 predicted targets supported by CLIP-Seq data, the CLIP-Seq data can explore anti-correlation (pearson correlation: r < 0, *p-value* < 0.05) between miRNA and target genes across diverse cancer types (more details can be found in Supplementary Table 1). (**B**) The representative CLIP-Seq data about anti-correlation between miR-133b and target genes. SUMO2 gene vs. miR-133b in Colon and Rectal adenocarcinoma (CRC) and CLTA gene vs. miR-133b in papillary thyroid carcinoma (THCA) were listed in here. (**C**) Enrichment analysis of KEGG pathway through Enricher, 13 top statistically enriched results was listed here, more details can be found in Supplementary Table 2. (**D**) Enrichment analysis of OMIM Disease through Enricher, 14 top statistically enriched results was listed here, more details can be found in Supplementary Table 2.

Because microRNA perform functions mainly depending on the way that miRNA suppresses the translation and stability of target genes by binding to the 3′-UTRs of target mRNAs, so first we focus on all target genes of miR-133b. In order to increase reliability, the experimentally validated genes (compiled from miRTarBase 6.0 [16], DIANA-TarBase v7.0 [17]) and predicted targets supported by CLIP-Seq data (compiled from starBase v2.0 [18]) were used in this review. As showed in Figure 2A, Figure 2B and Supplementary Table 1, 725 target genes eventually are selected. Enrichment analysis of KEGG pathway and OMIM Disease were performed through Enricher [19], the result showed that several pathway and diseases on cancer, including “Proteoglycans in cancer”, “Pathways in cancer”, “Central carbon metabolism in cancer”, “breast cancer” and “ovarian cancer”, are among the statistically enriched categories (Figure 2C, Figure 2D and Supplementary Table 2). The miRNA-mRNA interactions indicated miR-133b play a central role in human cancer.

### Modulatory effect of miR-133b in cancer cell proliferation and apoptosis

**Figure 3:**
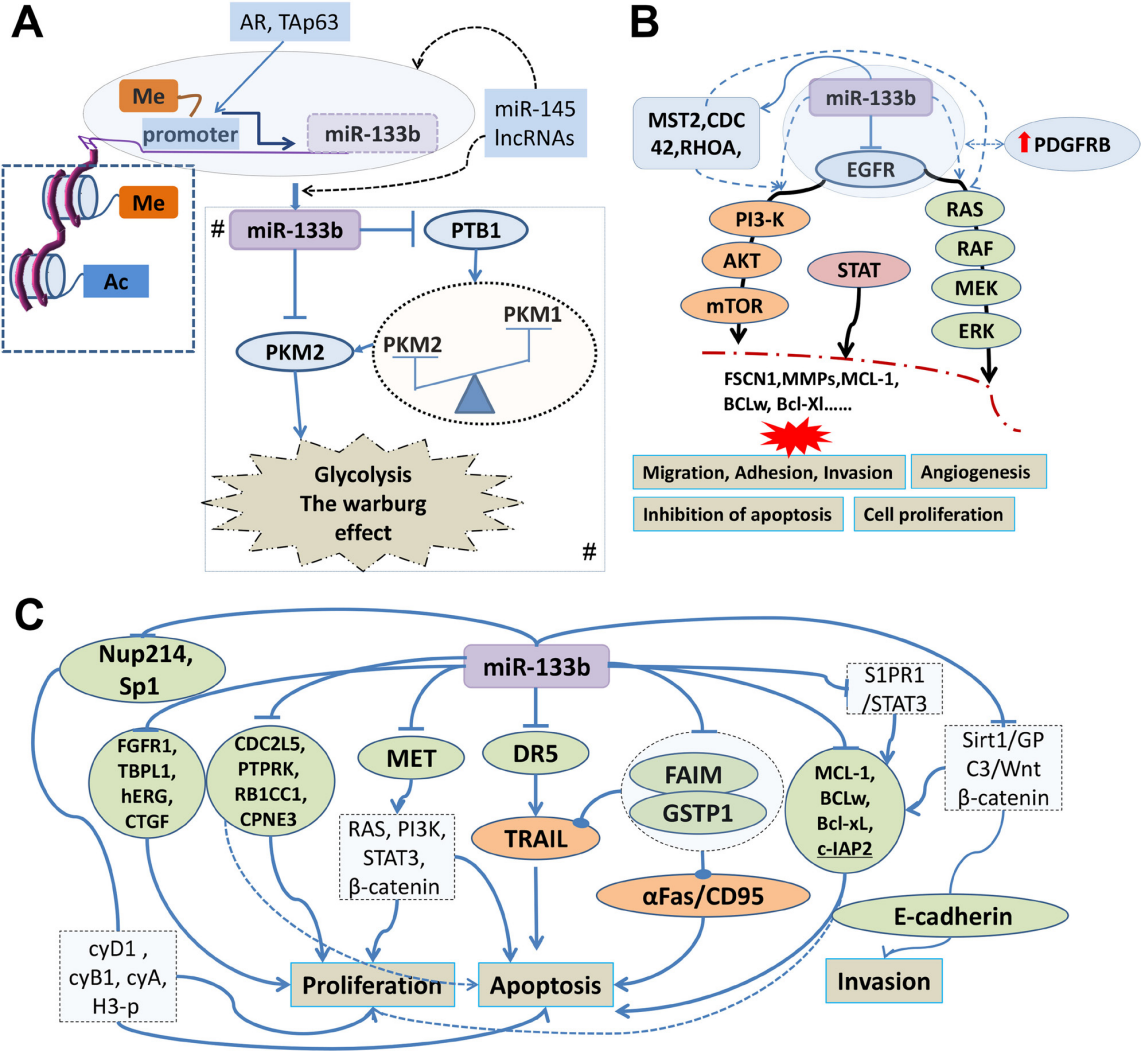
Tumor suppressive signatures of miR-133b involved in human cancer and its regulatory mechanism (**A**) The histone modification, promoter DNA hypermethylation and transcription factor including androgen receptor (AR) and tumor protein p63(TAp63) directly mediate miR-133b expression; miR-145 and Long non-coding RNAs (lncRNAs) may directly and indirectly regulate the expression of miR-133b. Me: methylation of histone and CpG island in promoter region. AC: acetylation; A# Regulatory network of miR-133b involved in altered energy metabolism of cancer cells. miR-133b control PKM expression (switching of PKM1 to PKM2) through targeting polypyrimidine tract-binding protein 1 (PTB1) or directly target PKM2 gene. (**B**) miR-133b can regulate the PI3K/AKT, MARK and STAT signaling pathways to participate in the occurrence and development of tumor by targeting epidermal growth factor receptor (EGFR), mammalian sterile 20-like kinase 2(MST2), cell division control protein 42 homolog (CDC42) or ras homolog gene family member A (RHOA); In glioblastoma (GBM) microvasculature, miR-133b, targeting and curbing EGFR indirectly elevated the expression of the platelet-derived growth factor receptor beta (PDGFRB). (**C**) Modulatory network of miR-133b in cancer cell proliferation, apoptosis and invasion. MiR-133b can participate in cancer cell proliferation; apoptosis and invasion through directly suppressing the gene including DR5, FAIM, GSTP1, CDC2L5, PTPRK, RB1CC1, CPNE3, MCL-1, BCL2L2, Mcl-1, Bcl-xL, FGFR1, Sp1, TBPL1, hERG, Kv11.1, KCNH2, CTGF, Nup214, c-Met, Sirt1 and S1PR1. These target genes directly or indirectly activate biological pathways to be involved in human cancer cell proliferation, apoptosis and invasion.

Sustaining proliferative signaling and resisting cell death are the most fundamental trait acquired during the cascade process of human tumors [20], the dysregulation of expression of miR-133b in cancers is often associated with these biological features via the deregulation of various validated gene target (Figure 3C), Table 1. Tumor necrosis-factor (TNF)-related apoptosis-inducing ligand (TRAIL) is a member of the TNF-superfamily that selectively induces apoptosis through death receptors (DRs) 4 and/or 5 in cancer cells, however, cancer cells can evade TRAIL-induced apoptosis by acquiring TRAIL-resistance [21]. Using α-mangostin, which is a xanthone derivative, to treat TRAIL-resistant human colon cancer DLD-1 cell line and breast epithelial proliferating MCF10A and found that α-mangostin curb the expression of miR-133b and relieve inhibition of miR-133b on its target gene DR5, which canceled the resistance and effectively induced the translocation of DR5 to the cancer cell surface membrane in TRAIL-resistant DLD-1 cells [22]. The difference is that miR-133b can caused exacerbated proapoptotic responses to TNF-related apoptosis-inducing ligand (TRAIL) in HeLa cells [23], what led to the phenomenon is miR-133b suppresses its immediate taget-antiapoptotic protein Fas apoptosis inhibitory molecule (FAIM) and antiapoptotic enzyme detoxifying protein glutathione-S-transferase pi (GSTP1) and promote TRAIL or αFas/CD95-mediated apoptosis, what is important is that the mechanism similar to HeLa cells can also be found in androgen-independent prostate cancer [23]. Interesting, miR-133b, recognized as androgen receptor (AR)targets, can promoting cell survival and proliferation in the androgen-dependent PCa cells by represses CDC2L5, PTPRK, RB1CC1, and CPNE3 [24], RB1CC1, which regulate cell growth, cell proliferation, apoptosis, can be repressed by miR-133b in less aggressive LNCaP prostate cancer cells [25]. Comparison of all these studies concludes that miR-133b have played a dual role in androgen-independent PCa and androgen-dependent PCa.

**Table 1:** The expression of miR-133b and its related targeting genes involved in human cancer

No.	Cancer types	Related gene	Desription	Function	Re.
1(+)	Hepatocellular carcinoma	PPP2R2D(−)	one of four isoforms (α, β, γ, and δ) of the protein phosphatase 2A	cell cycle regulation	[65]
2(−)	NSCLC (radioresistant)	PKM2(+)	pyruvate kinase isoform M2	inhibition of PKM2-mediated glycolysis	[40]
3(−)	Colorectal adenoma and cancer	PTB1;PKM2(+)/PKM1(−)	polypyrimidine tract-binding protein 1; Pyruvate kinase muscle has 2 isoforms	switch their PKM isoform from PKM1 to PKM2 and promotes the Warburg effect	[42]
4(−)	Ovarian cancer(chemotherapy resistance)	GST-π /MDR1(+)	glutathione S-transferase (GST)-π and multidrug resistance protein 1 (MDR1)	reduce ovarian cancer drug resistance	[69]
5(+)	glioblastoma (GMP)	EGFR (−)	epidermal growth factor receptor	microvascular proliferation	[52]
7(−)	Glioblastoma	MMP14)(+)	matrix metalloproteinase 14	inhibits cell migration and invasion	[56]
8(−)	Ovarian cancer	EGFR(+)	epidermal growth factor receptor	inhibit proliferation and invasion	[44]
9(+)	Cancer associated fibroblasts (CAF)	IL6 (untargeted gene)	interleukin-6	IL6-HPFs and (cancer associated fibroblasts) CAFs	[62]
10(?)	*DLD-1, #MCF10A cells	DR5(−)	death receptors 5	induces apoptosis	[18]
11(/)	(HeLa cells)cervical cancer	FAIM/GSTP1	antiapoptotic protein Fas apoptosis inhibitory molecule/detoxifying protein glutathione-S-transferase pi	induces apoptosis	[19]
12(−)	Androgen-independentprostate cancer	FAIM/GSTP1(+)	antiapoptotic protein Fas apoptosis inhibitory molecule/detoxifying protein glutathione-S-transferase pi	impaired proliferation and cellular metabolic activity and induces apoptosis	[19]
13(−)	Colorectal cancer	c-Met(+)	MET proto-oncogene, receptor tyrosine kinase	effect the proliferation and apoptosis	[33]
14(−)	Squamous cell carcinoma of tongue	PKM2(+)	pyruvate kinase isoform M2	interfere with the efficiency of proliferation and apoptosis	[43]
15(−)	Lung cancer	MCL-1/ BCL2L2 (BCLw)(+)	members of the B-cell CLL/lymphoma 2 (BCL-2) family of apoptotic molecules	induces apoptosis	[23]
16(−)	ESCC	FSCN1(+)	fascin actin-bundling protein 1	cell growth and invasion inhibition	[57]
17(+)	Cervical carcinoma	MST2/CDC42/RHOA(−)	mammalian sterile 20-like kinase 2/cell division control protein 42 homolog/ras homolog gene family member A	results in activation of AKT1 and ERK signaling pathways, and promotes both *in vivo* tumorigenesis and metastasis	[51]
18(−)	Prostate cancer	EGFR(+)	epidermal growth factor receptor	inhibited cell proliferation, migration and invasion	[45]
19(−)	Non-small cell lung cance	EGFR(+)	epidermal growth factor receptor	modulate apoptosis, invasion and sensitivity to EGFR-TKI	[47]
20(−)	Gastrointestinal stromal tumor	FSCN1(+)	fascin actin-bundling protein 1	tumor size, mitotic counts, risk grade, blood vessel invasion and mucosal ulceration	[58]
21(−)	Bladder cancer	EGRR(+)	epidermal growth factor receptor	inhibits cell proliferation, migration and invasion	[46]
22(−)	Osteosarcoma	MET(+)	MET proto-oncogene, receptor tyrosine kinase	control cell proliferation and cell cycle	[34]
23(−)	Gastric cancer	FGFR1(+)	fibroblast growth factor receptor-like 1	inhibit cell proliferation and colony formation	[27]
24(+)	Androgen-dependent Pca	CDC2L5, PTPRK, RB1CC1, and CPNE3(−)	encoded CDK13, tyrosine phosphatase (PTP) family, transcription factor termed RB1-inducible coiled-coil 1, calcium-dependent membrane-binding protein termed copine III	promoting cell survival and proliferation, basically required for mediating AR signalling to PCa cell viability and survival	[20]
25(−)	Colorectal cancer	CXCR4(+)	C-X-C motif chemokine receptor 4	inhibited invasion and stimulated apoptosis	[60]
25(−)	Osteosarcoma	BCL2L2, MCL-1, IGF1R, MET(+)	lymphoma 2 (BCL-2) family of apoptotic molecules, members of the B-cell CLL, insulin like growth factor 1 receptor, MET proto-oncogene, receptor tyrosine kinase	inhibited cell proliferation, invasion and migration, and induced apoptosis	[24]
26(−)	Gastric cancer	Gli1(+)	GLI family zinc finger 1, Zeb2 and OPN are direct transcriptional targets of Gli1	inhibits cell migration and invasion	[61]
27(−)	Colon cancer	RhoA, TAp63 (untargeted gene)(−)	ras homolog gene family member A, tumor protein p63, microRNA-133b is a transcriptional target of TAp63	TAp63 inhibits cell migration and invasion through microRNA-133b	[115]
28(+/–)	Prostate cancer	RB1CC1(−)	RB1 inducible coiled-coil 1	effect the cell proliferation, cell cycle, and apoptosis	[21]
29(−)	Colorectal cancer	TBPL1(+)	TATA-box binding protein like 1	proliferation	[29]
30(−)	Gastric cancer	Sp1(+)	Sp1 transcription factor	inhibit proliferation, migration, invasion and cell cycle progression	[28]
31(−)	Glioma	hERG, Kv11.1, KCNH2(+)	the human ether-a-go-go-related gene potassium channel	inhibition of proliferation of and induced apoptosis	[30]
32(−)	Bladder cancer	BCL2L2(Bcl-w), Akt1(+)	Bcl-2-like protein 2, serine/threonine kinase 1	inhibition of proliferation of and induced apoptosis	[25]
33(−)	Gastric cancer	FSCN1(+)	Fascin actin-bundling protein 1	inhibit cell proliferation, cell migration and invasion	[59]
34(−)	Several human cancer	Nup214(+)	nucleoporin 214	perturbs mitotic timing and leads to cell death	[32]
35(−)	Colorectal cancer	EGFR	epidermal growth factor receptor	inhibit growth and invasion	[48]
36(−)	Gastric cancer	Mcl-1, Bcl-xL(+)	BCL2 family apoptosis regulator	suppress GC cell proliferation and promote cell apoptosis	[26]
37(−)	Glioma	Sirt1(+)	silent information regulator 1	reduced the proliferation and invasion	[36]
38(−)	Hepatocellular carcinoma	Sirt1(+)	silent information regulator 1	regulating HCC cell proliferation, invasion and apoptosis	[35]
39(−)	Nasopharyngeal carcinoma	S1PR1(+)	Sphingosine-1-phosphate receptor 1	suppresses cell proliferation	[37]
40(−)	Hepatocellular carcinoma	CTGF(+)	Connective tissue growth factor	influences HCC cell proliferation and migration, and ductular reaction (DR)/oval cell (OC) response	[31]

HPF: human prostate fibroblasts;* DLD-1 cell line, TRAIL-resistant human colon cancer; # MCF10A cells, breast epithelial proliferating; -: decreased expression; +: increase expression. Re.: reference; GMP: Glioblastoma microvascular proliferation; ESCC: Esophageal squamous cell carcinoma

Besides TNF path, the Bcl-2 family is also important in regulate cell death (apoptosis), by either inducing (pro-apoptotic) or inhibiting (anti-apoptotic) apoptosis [26]. Two members of the BCL-2 family of pro-survival molecules (MCL-1 and BCL2L2 (BCLw)) as predicted targets of miR-133b have been identified in lung cancer, osteosarcoma, bladder cancer and gastric cancer (Mcl-1 and Bcl-xL), in which over-expression of miR-133b can induce apoptosis though theses apoptosis regulator in tumor cells [27–30]. Yet expect all the apoptotic pathways, miR-133b can regulate genes closely related to proliferation, such as fibroblast growth factor receptor 1 (FGFR1), and Sp1 and its downstream proteins Cyclin D1 in gastric cancer [31, 32], TATA-box binding protein like 1(TBPL1) in colorectal cancer [33], the human ether-a-go-go-related gene potassium channel (hERG, Kv11.1, KCNH2) in glioma [34]and connective tissue growth factor(CTGF) in hepatocellular carcinoma (HCC) [35].

What is particularly intriguing is that miR-133b can conspicuously suppress nucleoporin member Nup214 expression in serveral human cancer, and which increased mitotic indices and delayed degradation of mitotic marker proteins cyclinB1 and cyclinA and dephosphorylation of H3. Moreover, this mitotic delay boosts chromosomal abnormalities and apoptosis [36]. Some signaling pathway can be affected by miR-133b, such as c-Met engagement activates multiple oncogenic pathways (RAS, PI3K, STAT3, beta-catenin), which itself is the Immediate target gene of miR-133b in colorectal cancer (CC) and osteosarcoma, its suppression can affected tumor cell proliferation and apoptosis *in vitro* and *in vivo* [28, 37, 38]. In glioma and hepatocellular carcinoma, miR-133b inhibit its target gene silent information regulator 1 (Sirt1) and then suppress cell proliferation and invasion together with increasing apoptosis, the specific influence mechanism may be the miR-133b/Sirt1/GPC3/Wnt β-catenin pathway, by which a series of genes such as Bcl-2, Bcl-xL, Mcl-1 and E-cadherin were regulated [39, 40]. Anti-apoptotic oncogene Bcl-2, Mcl-1 and cellular inhibitor of apoptosis-2 (c-IAP2) also can be modulated through miR-133b /S1PR1 /STAT3 signaling in nasopharyngeal carcinoma, sphingosine-1-phosphate receptor 1 (S1PR1) was predicted to be a target of miR-133b [41].

### Regulatory role of miR-133b in altered energy metabolism of cancer cells

That metabolic alterations in cancer cells can drive tumor progression have already been widely accepted, a cancer-associated metabolic biochemistry pathway, Warburg effect, activates glycolysis and fuels tumor growth by providing energy and biosynthetic demands of cancer cells [42, 43]. In lung cancer cells, miR-133b suppresses glycolysis and improves radiotherapy by targeting PKM2 (pyruvate kinase isoform M2), which is an essential enzyme involved in glycolysis and promoted the Warburg effect [44]. Much evidence indicates that PKM1, another isoform of Pyruvate kinase muscle (PKM) gene, is expressed in normal differentiated tissues, whereas PKM2 is expressed in proliferating and cancer cells, heterogeneous nuclear ribonucleoprotein (hnRNP) family can regulate the alternative splicing to switch their PKM isoform from PKM1 to PKM2 during tumor development [45]. In colorectal adenoma cancer miR-133b controlling PKM expression (switching of PKM1 to PKM2) through targeting polypyrimidine tract-binding protein 1 (PTB1), which is a splicer of the PKM gene and also known as hnRNPI [46]. Earlier finding also showed that miR-133b was significantly reduced in squamous cell carcinoma (SCC) of tongue which depress PKM2 and interfere with the efficiency of proliferation and apoptosis [47]. (Figure 3A), Table 1.

### The miR-133b and epidermal growth factor receptor

The relationship between miR-133b and epidermal growth factor receptor (EGFR) separately discussed here mainly because the fact that miR-133b target EGFR or alter EGFR-specific genetic pathways have been found in several human cancer, such as ovarian cancer (OC) [48], prostate cancer (PC) [49], bladder cancer [50], (non-small-cell lung cancer) NSCLC [51], colorectal cancer [52]. And as we all know, EGFR, a transmembrane glycoprotein, constitutes one of the four members of ErbB family of tyrosine kinase receptors; activation of it can initiate several signal transduction cascades, principally the MAPK, Akt and JNK pathways [53], furthermore, EGFR overactivity have been associated with a number of cancers and led to the development of anticancer therapeutics called “EGFR inhibitors” [54]. It's sure that dysregulated miR-133b plays a crucial role in the process—then, what is the concrete mechanism? In OC, miR-133b can reduce the phosphorylation of Erk1/2 and Akt by targeting EGFR [48]. The protein level of EGFR, phosphorylated ERK and AKT, MMP-2 was significantly lowered in PC cell lines after transfection of miR-133a/b, which indicate miR-133a/b suppress EGFR in PC cells whereby inactivating the downstream signals, MMP-2, as an effector of EGFR pathway and mediating cell migration and invasion [49], similar mechanisms may occur in NSCLC [51]and in bladder cancer [50]. Equally attractive is combined treatment of miR-133b and cetuxima can intensify suppression effect on the growth and invasion of colorectal cancer cells by modulating EGFR [52]. Although not concerning the gene EGFR, upregulation of miR-133b in cervical carcinoma boosts tumorigenesis and metastasis by targeting mammalian sterile 20-like kinase 2 (MST2), cell division control protein 42 homolog (CDC42) and ras homolog gene family member A(RHOA), which subsequently bring about activation of the Akt and MARK signaling pathways [55].

Notwithstanding the fact that EGFR gene are commonly present in cancer, while there is a significant decrease in the expression of EGFR in glioblastoma (GBM) microvasculature, for the platelet-derived growth factor receptor beta (PDGFRB), involved in GBM angiogenesis, occupy the primary position in microvascular proliferation, one of the reason is several elevated expression of miRs, including miR-133b, targeting and curbing EGFR [56]. (Figure 3B), Table 1.

### Regulation mechanism of miR-133b inhibiting cell migration and innovation

The metastatic programme encompasses multiple sequential steps and involves a set of genes that regulate these complex processes. Note that many miRNAs are aberrantly expressed in various human cancers and regulate metastasis-associated genes and the vast majority of miRs performed functions by inhibit its downstream target genes [57, 58]. With the latest deciphering of roles for miR-133b in the metastatic programme there are numerous such cancer gene have been identified, such as MMP14, MMP-9, the member of MMPs family, can be suppressed in GBM and renal cell carcinoma [59, 60], respectively. (Figure 3B, 3C), Table 1. Fascin actin-bundling protein 1(FSCN1), which plays a critical role in cell migration, motility, adhesion and cellular interactionsin, in esophageal squamous cell carcinoma (ESCC) [61], gastrointestinal stromal tumor(GIST) [62] and gastric cancer [63], C-X-C motif chemokine receptor 4(CXCR4) and in colorectal cancer [64], IGF1R, MET, phospho-Akt and FAK in osteosarcoma [28], Gli1 in gastric cancer(GC) [65], Sp1 and its downstream proteins MMP-9 in GC [32], CTGF in HCC [35].

In addition, miR-133b can mediate cancer metastasis via regulating the tumor microenvironment, previous study [66] have found a marked up-regulation of miR-1 and miR-133b in interleukin-6 (IL6)-(human prostate fibroblasts) HPFs and (cancer associated fibroblasts) CAFs, miR-133b not only is able per se to promote fibroblast activation by inducing phenotypic changes but can be secreted by activated fibroblasts and may intake further by prostate carcinoma cells, by which mesenchymal phenotype would be established, and then CAFs induce EMT in tumor cells. These researches about miR-133b as metastasis promoter and metastasis suppressor therefore represent a new approach that may enhance our understanding of the molecular mechanisms modulating the metastatic cascade.

### Regulation mechanism of miR-133b in chemotherapy and radiotherapy

Chemotherapy still remains the mainstay of treatments for locally advanced and metastasized cancer, but chemo-resistant properties created insurmountable problems for cancer therapy. Recent clinical and experimental studys showed that dysregulation of specific miRNAs cause drug resistance in various cancers and modification of these miRNAs via miRNA mimics or antagomiRs can rehabilitate the gene regulatory network and sensitize cancerous cells to chemotherapy [67, 68].

B55δ, encoded by the PPP2R2D gene, can increase the suppressive effect of cisplatin (cDDP) in Hepatocellular carcinoma (HC), miR-133b was up-regulated in HC cells and directly target PPP2R2D and suppress its expression and further disrupt its effect in improving chemotherapeutic sensitivity [69]. Another study in HC [70], However, found that miR-133b showed downregulation and might act as tumour suppressor, then, the participants accepted transarterial chemoembolization (TACE) using chemotherapy agents-doxorubicin and cisplatin, miR-133b and othter 11 miRNAs were significantly upregulated in the patients group of nonresponders compared to responders, so research suggests 12 miRNAs might be cooperatively associated with the development of resistance to doxorubicin-cisplatin combined treatment, the underlying cause was that 3 miRNAs among theser miRNAs are directly linked to drug resistance in cancer, especially miR-27a and miR-130a can stimulate MDR1-mediated drug resistance in HC cells, it had been identified that multidrug resistance protein 1(MDR1 or ABCB1) involved in doxorubicin and cisplatin resistance [71, 72]. Similarity, yet paradoxically, miR-133b was significantly lower in primary resistant ovarian carcinomas and cell lines and reduced ovarian cancer drug resistance by silencing the expression of the drug-resistance-related proteins, glutathione S-transferase(GST-π) and multidrug resistance protein 1 (MDR1) [73]. Moreover, the response rate of esophageal squamous cell carcinoma (ESCC) patients to paclitaxel-based chemotherapy was significantly higher in combined miR-133a/b downregulation group [74], miR-133b contributes to arsenic-induced apoptosis in glioma cells [34] and the joint utilization of miR-133b and cetuxima can enhance suppression effect on the growth and invasion of colorectal cancer cells by modulating EGFR [52]. These studies, though present tissue specificity, clearly confirmed that the chemotherapy efficiency of cancer is more closely related to abnormal expression of miR-133b.

Besides the regulatory effects of miR-133b involved in chemosensitivity, some studies have also demonstrated its role playing in radiosensitivity. miR-133 was downregulated in radioresistant lung cancer cells, but restoring the miR-133b can resensitizes radioresistant lung cancer cells through the inhibition of PKM2-mediated glycolysis that interfere the sensitivity mechamism. Table 1.

### The expression of miR-133b

As discussed in the previous section, miR-133b performs functions mainly depending on target genes suppressed by it, so in the first place, all target genes of miR-133b involved in human cancer were reviewed above. But a lot of research only focuses on the altered expression of miR-133b itself in human cancer; these studies demonstrated important clinical significance but there are still a lot of great challenges or paradoxes for us to solve, especially the double expression level of miR-133b in different cancers and or even in single human cancer. In the sections that follow, the clinical implication of altered expression of miR-133b in human cancer and the possible problems or paradoxes were analyzed.

### Clinical implication of altered expression of miR-133b in human cancer

The identification of makers which can predict the oncogenesis and development of tumor is one of the highest priorities for translational cancer study. miRNAs could represent such much-anticipated biomakers and the potential for this had been identified for several different cancer forms [58, 75, 76]. To analyse and compare the pathological tissue by qPCR may be relatively commonly used in identifying promising tumor biomarkers, the overwhelming majority of the analyzed tumor entities showed significantly decreased intracellular miR-133b expression compared to matched non-malignant tissue, including lung cancer [77], urothelial carcinoma of bladder [78, 79], gastric cancer [63, 80], osteosarcoma [81]. These studies indicated that the downregulation of miR-133b was the independent factor and significantly associated with aggressive clinicopathologic features, tumor subtype, and poor survival rates. Similarity, qPCR evaluating microdissected preoperative biopsies from patients with rectal cancer showed a significant correlation between miR-133b and distant-metastasis-free survival [82]. In all resected colon cancer tissue without patient's recurrence, the miR-133b expression was significantly upregulated [83]. High expression of miR-185 and low expression of miR-133b were correlated with poor survival and metastasis in colorectal cancer [84], which suggest the potential prognostic values of these miRNAs for predicting clinical outcome after surgery, miR-1 and mir-133b have been significantly downregulated in recurrent PCa specimens and can serve as novel biomarkers for prediction of PCa progression [85]. Serum and secretions are another important clinical specimens, circulating serum miR-133b can be identified as the most important diagnostic and prognosis markers in breast cancer [86], gastric cancer [87] as well as in osteosarcoma [88], and miR-133b were significantly downregulated in prostatic secretions of patients with prostate cancer and could be used as diagnostics markers [89].

miR-133b may not only serve as both diagnostic and prognostic biomarkers but have great therapeutic potential in clinical practice. In a study of seeking the optimal miRNA delivery systems to treat lung cancer, miR-133b was selected because it directly targets the MCL-1 thus regulating cell survival and chemotherapeutic sensitivity, the result from this research demonstrated cationic lipoplexes may be a promising carrier system for miRNA-based therapeutics in lung cancer treatment [90]. Also in lung cancer, the experiment *in vivo* showed alteration of serum miR-206 and miR-133b may have to do with lung carcinogenesis induced by 4-(methylnitrosamino)-1-(3-pyridyl)-1-butanone [91]. MiR-133b consistently overexpressed in tolerogenic dendritic cells, which will provide possible therapeutic targets in the treatment of cancer and autoimmune diseases [92].

### The dual expression of miR-133b in human cancer

miRNA microarray data and most individual experiments have demonstrated that miR-133b was frequently down-regulated in various cancers and have tumor-suppressive functions [93], such as firstly been detected in colorectal cancer [64, 94, 95], and subsequently in SCC of tongue [47, 96], bladder cancer [97, 98], urothelial carcinoma of the bladder [78, 99, 100], lung cancer [51, 77, 101], glioblastoma [34, 60], ovarian cancer [48], prostate cancer [23, 49], gastric cancer [31, 80, 99, 102, 103], head and neck cancer [104], GIST [62], osteosarcoma [38, 105], rhabdomyosarcomas [106], ESCC [61, 102, 103, 107], uterine sarcomas and mixed epithelial-mesenchymal uterine tumors [108], renal cell carcinoma [109], hepatocellular carcinoma [39], laryngeal cancer [110]. Some cancers however show that elevation of miR-133b levels promotes cancer progression. Such as miR-133b with a high expression in HC, the miR-133b/PPP2R2D signaling pathway affects the effectiveness of cDDP chemotherapy [69], upregulation of miR-133b shortens the latency of cervical carcinoma [55, 111], miR-133b is directly up-regulated by AR in androgen-dependent PCa [24], overexpression of miR-133b as VHL-specific miRNAs in pheochromocytoma and paraganglioma [112], Overexpression of miR-133b in less aggressive LNCaP cells boosted cell proliferation and cell-cycle progression [25] and have decreased survival in progression bladder cancer [79], compared to primary colorectal tumors, the cases with liver metastases demonstrated increased expression of miR-210 and miR-133b and associated with lower survival [113]. Moreover, microenvironment associated with cancer also have high expression of miR-133b, such as miR-133b in cancer associated fibroblasts [66], in GBM microvasculature [56]. Yet it is also worth noting that different research teams shows distinctly different miR-133b profiles even in the same tissues, the reason leading to these potentially conflicting and inconsistent results may be the method of miR detection, different intracellular signal transduction pathway, different miR assay platforms or the difficulties to distinguish the mature miR isomers (miR-133b belongs to myomiR families).

**Figure 4:**
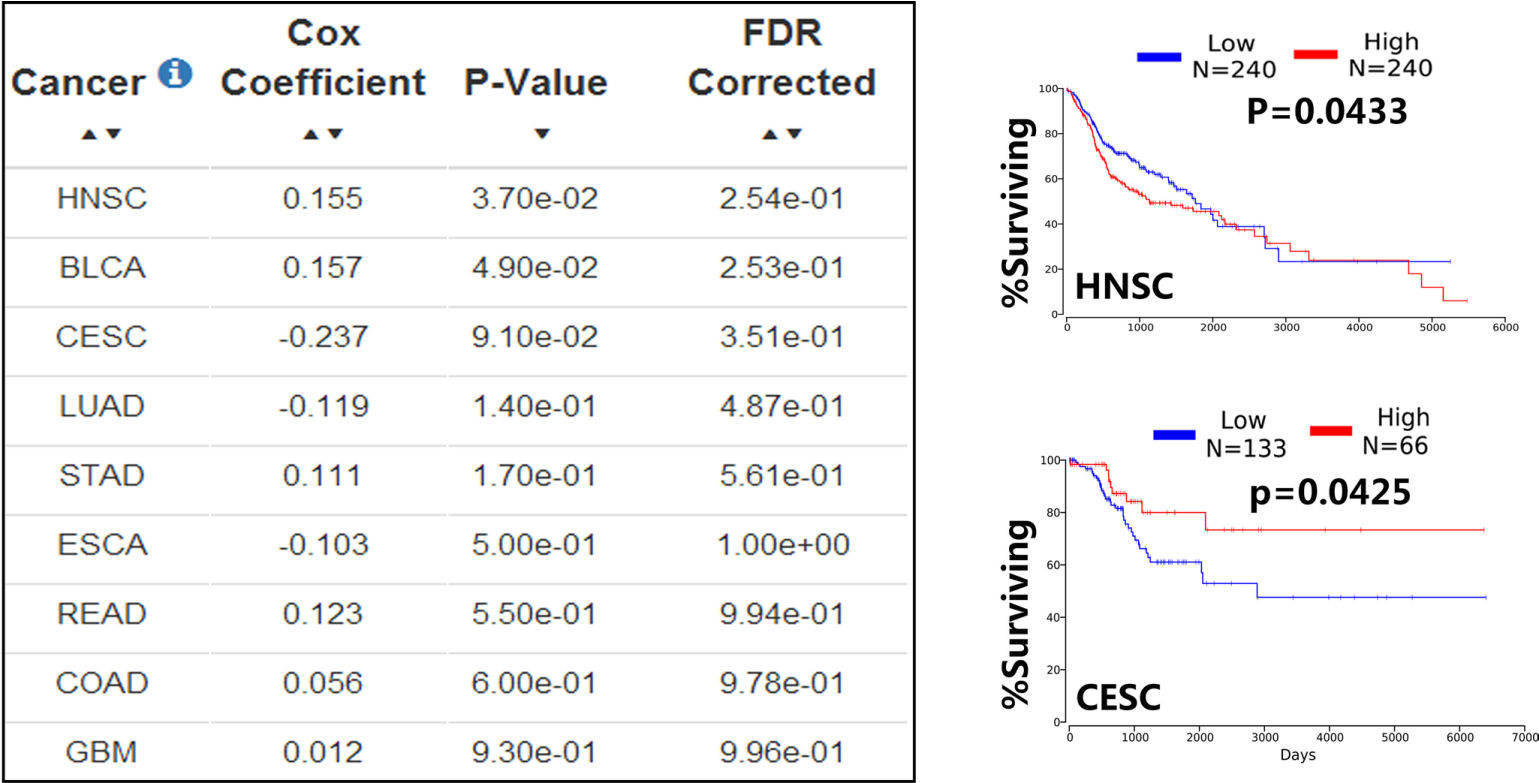
The dual expression of miR-133b in human cancer The database OncoLnc was used to explore survival correlations of miR-133b in cancer, the database provided 9 cancer data between miR-133b and human cancer, the Cox coefficient and *p-value* are from the gene term in precomputed multivariate Cox regressions and the FDR correction is performed per cancer analysis per data type (left); the representative example of OncoLnc Kaplan-Meier results(right), in head and neck squamous cell carcinoma(HNSC), the low expression of miR-133b have a higher survival rate(upper right); in Cervical cancer (CESC), the high expression of miR-133b have a higher survival rate(lower right). More details and abbreviation can be found in TCGA(https://cancergenome.nih.gov/) and OncoLnc(http://www.oncolnc.org/).

To better understand the role of altered expression of miR-133b in human cancer, we use OncoLnc (http://www.oncolnc.org/) exploring survival correlations of miR-133b in cancer (OncoLnc is a database can provide survival data for 8,647 patients from 21 cancer studies performed by The Cancer Genome Atlas (TCGA)). The result showed 9 cancer data which uncovered similar results as above that different expression of miR-133b plays tumor suppressor and tumor promoter in various malignant tumors. (Figure 4). All indicated the fact that miR-133b regarded as much-anticipated biomarkers is gradually moving, though much is still to be done.

### Regulatory mechanisms leading to abnormal expression of miR-133b

What matters, and become more particularly significant in all these studies, is the abnormally expressed miR-133b in human cancer and to illuminates the underlying mechanism resulting in alteration. Regulation of microRNA expression can be exerted through several mechanisms such as chromosomal abnormalities, biogenesis defects, epigenetic changes, transcription, miRs-oncogenes feedback mechanism [4, 114].

### Epigenetic modifications

Epigenetics is a pivotal mechanism involved in regulating the progression of malignant tumor. Existing studies have shown that miR-133b dysregulation in tumor is regulated by epigenetic modifications. Histone methylation inhibitor DZNep or histone deacetylation (HDAC) inhibitor SAHA consistently increased the expression of miR-133b/a-3p in GC cell lines, and last but not least, decreased levels of H3 acetylation and increased levels of H3K27me3 were observed in the promoter region of miR-133b, all this indicating that both histone methylation and histone acetylation might be responsible for miR-133b/a-3p downregulation, but simultaneously discover showed DNA methylation inhibitor 5-Azacytidine't didn't increased the expression of miR-133b/a-3p in GC cell lines [30]. Compared with it, a research conducted in colorectal cancer found the CpG islands of miR-133b promoter presents hypermethylation in CRC tissues and cells, functional analysis demonstrated that demethylation treatment with 5-aza-2'-deoxycytidine (5-Aza-CdR) can increased the expression of miR-133b and then reversed its anticancer effects. At the same time, the study also shows that histone deacetylase (HDAC) inhibitor (PBA) can increase the expression of silenced miR-133b, which may imply histone modification also mediate miR-133b expression [115].

In addition, miRs themselves are capable of targeting genes which regulate epigenetic pathways. Dysregulation of DNMTs, a key DNA methylation enzyme including DNMTs 1, 3A, and 3B, has been linked to various disease processes, including cancer and congenital disorders. These enzymes are predicted to be potential targets of miRNAs, just as the miR-29 can regulate methylation-silenced genes by target DNMTs 3A and 3B. But in a research of acute myeloid leukemia, Niederwieser C et al. [116] found that high DNMT3B expression seemingly an independent prognostic factor from mechanisms of DNA hypermethylation and/or microRNA-dependent gene repression, at the same time they attempted to explain why the miR-133b presented unique upregulation in high DNMT3B expressers. In summary, the epigenetics–miRNA regulatory circuit plays an important role in abnormal expression of miR-133b. (Figure 3A)

### Transcription factor and noncoding RNAs involved regulation

The dysregulated miRs expression in cancer can also result from increased or decreased transcription activity at the gene promoter. Some reports in prostate cancer have identified miRs in androgen receptor (AR) signaling, such as miR-21, owing to AR binding on the defined promoter, was directly up-regulated by AR [117]. To better elucidate miRs roles in AR signaling, Wenjuan Mo etc. [24] introduced Response Score to identify AR target miRNAs and 15 miRNAs were theoretically identified as candidate in the end, based on GenMAPP and ChIP assay results they found a significant AR-binding to the chromatin of predicted AREs (in the upstream and downstream 15 kb of pre-miRNA's 5′-start site) in miR-19a, miR-27a and miR-133b in treated LNCaP cells, and AR driven transcription of these miRs. An extensive new research study [118] has found that flavin-dependent monoamine oxidase KDM1A can triggers androgen-induced miR-133b transcription via H3K4me2 demethylation and DNA oxidation. Similarly, miR-133b dysregulation in colon cancer cells was allegedly due to the change of transcription activity caused by TAp63, miR-133b is a transcriptional target of TAp63 so that p63 can directly drives the miR-133b expression via the binding site, what is important is that TAp63 can inhibit cells migration and metastasis by indirectly regulating the expression of target gene of miR-133b (such as RhoA) and epithelial–mesenchymal markers in colon cancer in virtue of TAp63/miR-133b axis [119].

In addition to changes of transcription activity of a transcription factor, microRNA expression can be also modulated as a consequence of potential synergy between noncoding RNAs, such as miRs themself and long non-coding RNA, a research that miRNA profiling and quantitative integrated omics analysis of a pancreatic cancer cell line found overexpression of miR-145 can increase other miRs including miR-124, miR-133b and miR-125a-3p, all of which are implicated in inhibition of tumors and are generally co-regulated with miR-145 in other cancers [120]. Long non-coding RNAs (lncRNAs) have emerged in recent years engaging in numerous biological processes across species [121]; despite no researches about miR-133b-lncRNA regulatory network have been reported in human cancer, but the research evidence from out group (unpublished) and the fact that miR-133b is one of the earliest validated ceRNA regulators [122] make us believe that the mutual regulation and synergistic effects of miR-133b-lncRNA can be found in tumor. (Figure 3A).

### Conclusion and perspective

As noted above, discrepancies present in miR-133b profiles detecting, even in same tissues. One the underlying causes may be that most of these researches come from individual studies and small sample size, despite the ethnic variation and tissue specificity, more researches need to be done and techniques just as employing pri- or pre-miR RT-PCR assays and deep sequencing should be improved and adopted to accurately re-evaluate the genome-wide miRs expression profiles of different cancers. Simultaneously, miRs-mRNAs regulatory networks are extraordinarily complex; some research indicates that only a small portion of protein expression changes could be explained by predicted direct binding of miRNAs to corresponding mRNAs [123], so more detailed endogenous network regulation should be clarified. All these are important to understand the regulation process of particular pathological states. In the era of personalized medicine, miRNAs can become a long-awaited and was full of prospects marker in diagnostic and therapy development for various kinds of human cancers. Nonetheless, there are still lots of new challenging jobs to address in order to take advantage of miRNAs as efficient and safe therapeutics.

Here, we describe miR-133b, one of the myomiRs, was involved in human cancer, stressing its individualistic roles as tumor activators and suppressors, and discusses the concrete mechanism playing in different hallmarks of cancer and possible use in the clinic as predictive markers and as therapeutic strategies for tumor patients. With the in-depth research, individual myomiRs have unique functional roles in the physiological-biochemical mechanism of numerous non-muscle cells and tissues, beyond their initial definition as muscle-specific factors, and hence elucidation of these myomiRs and their complicated regulation network together or independently becomes particularly important.

## SUPPLEMENTARY MATERIALS







## References

[1] Iorio MV, Croce CM (2012). Causes and consequences of microRNA dysregulation. Cancer J.

[2] Bartel DP (2004). MicroRNAs: genomics, biogenesis, mechanism, and function. Cell.

[3] Wu WK, Lee CW, Cho CH, Fan D, Wu K, Yu J, Sung JJ (2010). MicroRNA dysregulation in gastric cancer: a new player enters the game. Oncogene.

[4] Lin S, Gregory RI (2015). MicroRNA biogenesis pathways in cancer. Nat Rev Cancer.

[5] Nohata N, Hanazawa T, Enokida H, Seki N (2012). microRNA-1/133a and microRNA-206/133b clusters: dysregulation and functional roles in human cancers. Oncotarget.

[6] Mitchelson KR, Qin WY (2015). Roles of the canonical myomiRs miR-1, -133 and -206 in cell development and disease. World J Biol Chem.

[7] Yu H, Lu Y, Li Z, Wang Q (2014). microRNA-133: expression, function and therapeutic potential in muscle diseases and cancer. Curr Drug Targets.

[8] Townley-Tilson WH, Callis TE, Wang D (2010). MicroRNAs 1, 133, and 206: critical factors of skeletal and cardiac muscle development, function, and disease. Int J Biochem Cell Biol.

[9] Horak M, Novak J, Bienertova-Vasku J (2016). Muscle-specific microRNAs in skeletal muscle development. Dev Biol.

[10] Chiba Y, Misawa M (2010). MicroRNAs and their therapeutic potential for human diseases: MiR-133a and bronchial smooth muscle hyperresponsiveness in asthma. J Pharmacol Sci.

[11] Han C, Yu Z, Duan Z, Kan Q (2014). Role of microRNA-1 in human cancer and its therapeutic potentials. Biomed Res Int.

[12] Li J, Dong X, Wang Z, Wu J (2014). MicroRNA-1 in Cardiac Diseases and Cancers. Korean J Physiol Pharmacol.

[13] Weiss M, Brandenburg LO, Burchardt M, Stope MB (2016). MicroRNA-1 properties in cancer regulatory networks and tumor biology. Crit Rev Oncol Hematol.

[14] Ma G, Wang Y, Li Y, Cui L, Zhao Y, Zhao B, Li K (2015). MiR-206, a key modulator of skeletal muscle development and disease. Int J Biol Sci.

[15] McCarthy JJ (2008). MicroRNA-206: the skeletal muscle-specific myomiR. Biochim Biophys Acta.

[16] Chou CH, Chang NW, Shrestha S, Hsu SD, Lin YL, Lee WH, Yang CD, Hong HC, Wei TY, Tu SJ, Tsai TR, Ho SY, Jian TY (2016). miRTarBase 2016: updates to the experimentally validated miRNA-target interactions database. Nucleic Acids Res.

[17] Vlachos IS, Paraskevopoulou MD, Karagkouni D, Georgakilas G, Vergoulis T, Kanellos I, Anastasopoulos IL, Maniou S, Karathanou K, Kalfakakou D, Fevgas A, Dalamagas T, Hatzigeorgiou AG (2015). DIANA-TarBase v7.0: indexing more than half a million experimentally supported miRNA: mRNA interactions. Nucleic Acids Res.

[18] Li JH, Liu S, Zhou H, Qu LH, Yang JH (2014). starBase v2.0: decoding miRNA-ceRNA, miRNA-ncRNA and protein-RNA interaction networks from large-scale CLIP-Seq data. Nucleic Acids Res.

[19] Kuleshov MV, Jones MR, Rouillard AD, Fernandez NF, Duan Q, Wang Z, Koplev S, Jenkins SL, Jagodnik KM, Lachmann A, McDermott MG, Monteiro CD, Gundersen GW (2016). Enrichr: a comprehensive gene set enrichment analysis web server 2016 update. Nucleic Acids Res.

[20] Hanahan D, Weinberg RA (2011). Hallmarks of cancer: the next generation. Cell.

[21] Jung EM, Park JW, Choi KS, Park JW, Lee HI, Lee KS, Kwon TK (2006). Curcumin sensitizes tumor necrosis factor-related apoptosis-inducing ligand (TRAIL)-mediated apoptosis through CHOP-independent DR5 upregulation. Carcinogenesis.

[22] Kumazaki M, Shinohara H, Taniguchi K, Ueda H, Nishi M, Ryo A, Akao Y (2015). Understanding of tolerance in TRAIL-induced apoptosis and cancelation of its machinery by alpha-mangostin, a xanthone derivative. Oncotarget.

[23] Patron JP, Fendler A, Bild M, Jung U, Muller H, Arntzen MO, Piso C, Stephan C, Thiede B, Mollenkopf HJ, Jung K, Kaufmann SH, Schreiber J (2012). MiR-133b targets antiapoptotic genes and enhances death receptor-induced apoptosis. PLoS One.

[24] Mo W, Zhang J, Li X, Meng D, Gao Y, Yang S, Wan X, Zhou C, Guo F, Huang Y, Amente S, Avvedimento EV, Xie Y, Li Y (2013). Identification of novel AR-targeted microRNAs mediating androgen signalling through critical pathways to regulate cell viability in prostate cancer. PLoS One.

[25] Li X, Wan X, Chen H, Yang S, Liu Y, Mo W, Meng D, Du W, Huang Y, Wu H, Wang J, Li T, Li Y (2014). Identification of miR-133b and RB1CC1 as independent predictors for biochemical recurrence and potential therapeutic targets for prostate cancer. Clin Cancer Res.

[26] Cleary ML, Smith SD, Sklar J (1986). Cloning and structural analysis of cDNAs for bcl-2 and a hybrid bcl-2/immunoglobulin transcript resulting from the t(14;18) translocation. Cell.

[27] Crawford M, Batte K, Yu L, Wu X, Nuovo GJ, Marsh CB, Otterson GA, Nana-Sinkam SP (2009). MicroRNA 133B targets pro-survival molecules MCL-1 and BCL2L2 in lung cancer. Biochem Biophys Res Commun.

[28] Zhao H, Li M, Li L, Yang X, Lan G, Zhang Y (2013). MiR-133b is down-regulated in human osteosarcoma and inhibits osteosarcoma cells proliferation, migration and invasion, and promotes apoptosis. PLoS One.

[29] Chen XN, Wang KF, Xu ZQ, Li SJ, Liu Q, Fu DH, Wang X, Wu B (2014). MiR-133b regulates bladder cancer cell proliferation and apoptosis by targeting Bcl-w and Akt1. Cancer Cell Int.

[30] Liu Y, Zhang X, Zhang Y, Hu Z, Yang D, Wang C, Guo M, Cai Q (2015). Identification of miRNomes in human stomach and gastric carcinoma reveals miR-133b/a-3p as therapeutic target for gastric cancer. Cancer Lett.

[31] Wen D, Li S, Ji F, Cao H, Jiang W, Zhu J, Fang X (2013). miR-133b acts as a tumor suppressor and negatively regulates FGFR1 in gastric cancer. Tumour Biol.

[32] Qiu T, Zhou X, Wang J, Du Y, Xu J, Huang Z, Zhu W, Shu Y, Liu P (2014). MiR-145, miR-133a and miR-133b inhibit proliferation, migration, invasion and cell cycle progression via targeting transcription factor Sp1 in gastric cancer. FEBS Lett.

[33] Xiang KM, Li XR (2014). MiR-133b acts as a tumor suppressor and negatively regulates TBPL1 in colorectal cancer cells. Asian Pac J Cancer Prev.

[34] Wang J, Li Y, Jiang C (2015). MiR-133b contributes to arsenic-induced apoptosis in U251 glioma cells by targeting the hERG channel. J Mol Neurosci.

[35] Gjymishka A, Pi L, Oh SH, Jorgensen M, Liu C, Protopapadakis Y, Patel A, Petersen BE (2016). miR-133b Regulation of Connective Tissue Growth Factor: A Novel Mechanism in Liver Pathology. Am J Pathol.

[36] Bhattacharjya S, Roy KS, Ganguly A, Sarkar S, Panda CK, Bhattacharyya D, Bhattacharyya NP, Roychoudhury S (2015). Inhibition of nucleoporin member Nup214 expression by miR-133b perturbs mitotic timing and leads to cell death. Mol Cancer.

[37] Hu G, Chen D, Li X, Yang K, Wang H, Wu W (2010). miR-133b regulates the MET proto-oncogene and inhibits the growth of colorectal cancer cells in vitro and in vivo. Cancer Biol Ther.

[38] Novello C, Pazzaglia L, Cingolani C, Conti A, Quattrini I, Manara MC, Tognon M, Picci P, Benassi MS (2013). miRNA expression profile in human osteosarcoma: role of miR-1 and miR-133b in proliferation and cell cycle control. Int J Oncol.

[39] Tian Z, Jiang H, Liu Y, Huang Y, Xiong X, Wu H, Dai X (2016). MicroRNA-133b inhibits hepatocellular carcinoma cell progression by targeting Sirt1. Exp Cell Res.

[40] Li C, Liu Z, Yang K, Chen X, Zeng Y, Liu J, Li Z, Liu Y (2016). miR-133b inhibits glioma cell proliferation and invasion by targeting Sirt1. Oncotarget.

[41] Cheng N, Wang GH (2016). miR-133b, a microRNA targeting S1PR1, suppresses nasopharyngeal carcinoma cell proliferation. Exp Ther Med.

[42] Tasselli L, Chua KF (2012). Cancer: Metabolism in ‘the driver's seat. Nature.

[43] Seton-Rogers S (2016). Tumour metabolism: Adapting to harsh conditions. Nat Rev Cancer.

[44] Liu G, Li YI, Gao X (2016). Overexpression of microRNA-133b sensitizes non-small cell lung cancer cells to irradiation through the inhibition of glycolysis. Oncol Lett.

[45] Chen M, Zhang J, Manley JL (2010). Turning on a fuel switch of cancer: hnRNP proteins regulate alternative splicing of pyruvate kinase mRNA. Cancer Res.

[46] Taniguchi K, Ito Y, Sugito N, Kumazaki M, Shinohara H, Yamada N, Nakagawa Y, Sugiyama T, Futamura M, Otsuki Y, Yoshida K, Uchiyama K, Akao Y (2015). Organ-specific PTB1-associated microRNAs determine expression of pyruvate kinase isoforms. Sci Rep.

[47] Wong TS, Liu XB, Chung-Wai Ho A, Po-Wing Yuen A, Wai-Man Ng R, Ignace Wei W (2008). Identification of pyruvate kinase type M2 as potential oncoprotein in squamous cell carcinoma of tongue through microRNA profiling. Int J Cancer.

[48] Liu X, Li G (2015). MicroRNA-133b inhibits proliferation and invasion of ovarian cancer cells through Akt and Erk1/2 inactivation by targeting epidermal growth factor receptor. Int J Clin Exp Pathol.

[49] Tao J, Wu D, Xu B, Qian W, Li P, Lu Q, Yin C, Zhang W (2012). microRNA-133 inhibits cell proliferation, migration and invasion in prostate cancer cells by targeting the epidermal growth factor receptor. Oncol Rep.

[50] Zhou Y, Wu D, Tao J, Qu P, Zhou Z, Hou J (2013). MicroRNA-133 inhibits cell proliferation, migration and invasion by targeting epidermal growth factor receptor and its downstream effector proteins in bladder cancer. Scand J Urol.

[51] Liu L, Shao X, Gao W, Zhang Z, Liu P, Wang R, Huang P, Yin Y, Shu Y (2012). MicroRNA-133b inhibits the growth of non-small-cell lung cancer by targeting the epidermal growth factor receptor. FEBS J.

[52] Zhou J, Lv L, Lin C, Hu G, Guo Y, Wu M, Tian B, Li X (2015). Combinational treatment with microRNA133b and cetuximab has increased inhibitory effects on the growth and invasion of colorectal cancer cells by regulating EGFR. Mol Med Rep.

[53] Oda K, Matsuoka Y, Funahashi A, Kitano H (2005). A comprehensive pathway map of epidermal growth factor receptor signaling. Mol Syst Biol.

[54] Paez JG, Janne PA, Lee JC, Tracy S, Greulich H, Gabriel S, Herman P, Kaye FJ, Lindeman N, Boggon TJ, Naoki K, Sasaki H, Fujii Y (2004). EGFR mutations in lung cancer: correlation with clinical response to gefitinib therapy. Science.

[55] Qin W, Dong P, Ma C, Mitchelson K, Deng T, Zhang L, Sun Y, Feng X, Ding Y, Lu X, He J, Wen H, Cheng J (2012). MicroRNA-133b is a key promoter of cervical carcinoma development through the activation of the ERK and AKT1 pathways. Oncogene.

[56] Xu G, Li JY (2016). Differential expression of PDGFRB and EGFR in microvascular proliferation in glioblastoma. Tumour Biol.

[57] Aigner A (2011). MicroRNAs (miRNAs) in cancer invasion and metastasis: therapeutic approaches based on metastasis-related miRNAs. J Mol Med (Berl).

[58] Bouyssou JM, Manier S, Huynh D, Issa S, Roccaro AM, Ghobrial IM (2014). Regulation of microRNAs in cancer metastasis. Biochim Biophys Acta.

[59] Wu D, Pan H, Zhou Y, Zhou J, Fan Y, Qu P (2014). microRNA-133b downregulation and inhibition of cell proliferation, migration and invasion by targeting matrix metallopeptidase-9 in renal cell carcinoma. Mol Med Rep.

[60] Chang L, Lei X, Qin YU, Zhang X, Jin H, Wang C, Wang X, Li G, Tan C, Su J (2015). MicroRNA-133b inhibits cell migration and invasion by targeting matrix metalloproteinase 14 in glioblastoma. Oncol Lett.

[61] Kano M, Seki N, Kikkawa N, Fujimura L, Hoshino I, Akutsu Y, Chiyomaru T, Enokida H, Nakagawa M, Matsubara H (2010). miR-145, miR-133a and miR-133b: Tumor-suppressive miRNAs target FSCN1 in esophageal squamous cell carcinoma. Int J Cancer.

[62] Yamamoto H, Kohashi K, Fujita A, Oda Y (2013). Fascin-1 overexpression and miR-133b downregulation in the progression of gastrointestinal stromal tumor. Mod Pathol.

[63] Guo L, Bai H, Zou D, Hong T, Liu J, Huang J, He P, Zhou Q, He J (2014). The role of microRNA-133b and its target gene FSCN1 in gastric cancer. J Exp Clin Cancer Res.

[64] Duan FT, Qian F, Fang K, Lin KY, Wang WT, Chen YQ (2013). miR-133b, a muscle-specific microRNA, is a novel prognostic marker that participates in the progression of human colorectal cancer via regulation of CXCR4 expression. Mol Cancer.

[65] Zhao Y, Huang J, Zhang L, Qu Y, Li J, Yu B, Yan M, Yu Y, Liu B, Zhu Z (2014). MiR-133b is frequently decreased in gastric cancer and its overexpression reduces the metastatic potential of gastric cancer cells. BMC Cancer.

[66] Doldi V, Callari M, Giannoni E, D'Aiuto F, Maffezzini M, Valdagni R, Chiarugi P, Gandellini P, Zaffaroni N (2015). Integrated gene and miRNA expression analysis of prostate cancer associated fibroblasts supports a prominent role for interleukin-6 in fibroblast activation. Oncotarget.

[67] Kong YW, Ferland-McCollough D, Jackson TJ, Bushell M (2012). microRNAs in cancer management. Lancet Oncol.

[68] Fanini F, Fabbri M (2016). MicroRNAs and cancer resistance: A new molecular plot. Clin Pharmacol Ther.

[69] Zhuang Q, Zhou T, He C, Zhang S, Qiu Y, Luo B, Zhao R, Liu H, Lin Y, Lin Z (2016). Protein phosphatase 2A-B55delta enhances chemotherapy sensitivity of human hepatocellular carcinoma under the regulation of microRNA-133b. J Exp Clin Cancer Res.

[70] El-Halawany MS, Ismail HM, Zeeneldin AA, Elfiky A, Tantawy M, Kobaisi MH, Hamed I, Abdel Wahab AH. (2015). Investigating the pretreatment miRNA expression patterns of advanced hepatocellular carcinoma patients in association with response to TACE treatment. Biomed Res Int.

[71] Chen Z, Ma T, Huang C, Zhang L, Lv X, Xu T, Hu T, Li J (2013). MiR-27a modulates the MDR1/P-glycoprotein expression by inhibiting FZD7/beta-catenin pathway in hepatocellular carcinoma cells. Cell Signal.

[72] Xu N, Shen C, Luo Y, Xia L, Xue F, Xia Q, Zhang J (2012). Upregulated miR-130a increases drug resistance by regulating RUNX3 and Wnt signaling in cisplatin-treated HCC cell. Biochem Biophys Res Commun.

[73] Chen S, Jiao JW, Sun KX, Zong ZH, Zhao Y (2015). MicroRNA-133b targets glutathione S-transferase pi expression to increase ovarian cancer cell sensitivity to chemotherapy drugs. Drug Des Devel Ther.

[74] Chen G, Peng J, Zhu W, Tao G, Song Y, Zhou X, Wang W (2014). Combined downregulation of microRNA-133a and microRNA-133b predicts chemosensitivity of patients with esophageal squamous cell carcinoma undergoing paclitaxel-based chemotherapy. Med Oncol.

[75] Nicoloso MS, Spizzo R, Shimizu M, Rossi S, Calin GA (2009). MicroRNAs--the micro steering wheel of tumour metastases. Nat Rev Cancer.

[76] Cristobal I, Madoz-Gurpide J, Martin-Aparicio E, Carames C, Aguilera O, Rojo F, Garcia-Foncillas J (2014). Comment on ‘TAp63 suppress metastasis via miR-133b in colon cancer cells’. Br J Cancer.

[77] Chen SW, Wang TB, Tian YH, Zheng YG (2015). Down-regulation of microRNA-126 and microRNA-133b acts as novel predictor biomarkers in progression and metastasis of non small cell lung cancer. Int J Clin Exp Pathol.

[78] Chen X, Wu B, Xu Z, Li S, Tan S, Liu X, Wang K (2016). Downregulation of miR-133b predict progression and poor prognosis in patients with urothelial carcinoma of bladder. Cancer Med.

[79] Dyrskjot L, Ostenfeld MS, Bramsen JB, Silahtaroglu AN, Lamy P, Ramanathan R, Fristrup N, Jensen JL, Andersen CL, Zieger K, Kauppinen S, Ulhoi BP, Kjems J (2009). Genomic profiling of microRNAs in bladder cancer: miR-129 is associated with poor outcome and promotes cell death in vitro. Cancer Res.

[80] Chen Z, Liu X, Hu Z, Wang Y, Liu M, Liu X, Li H, Ji R, Guo Q, Zhou Y (2015). Identification and characterization of tumor suppressor and oncogenic miRNAs in gastric cancer. Oncol Lett.

[81] Bassampour SA, Abdi R, Bahador R, Shakeri M, Torkaman A, Yahaghi E, Taheriazam A (2015). Downregulation of miR-133b/miR-503 acts as efficient prognostic and diagnostic factors in patients with osteosarcoma and these predictor biomarkers are correlated with overall survival. Tumour Biol.

[82] Azizian A, Epping I, Kramer F, Jo P, Bernhardt M, Kitz J, Salinas G, Wolff HA, Grade M, Beissbarth T, Ghadimi BM, Gaedcke J (2016). Prognostic Value of MicroRNAs in Preoperative Treated Rectal Cancer. Int J Mol Sci.

[83] Tanoglu A, Balta AZ, Berber U, Ozdemir Y, Emirzeoglu L, Sayilir A, Sucullu I (2015). MicroRNA expression profile in patients with stage II colorectal cancer: a Turkish referral center study. Asian Pac J Cancer Prev.

[84] Akcakaya P, Ekelund S, Kolosenko I, Caramuta S, Ozata DM, Xie H, Lindforss U, Olivecrona H, Lui WO (2011). miR-185 and miR-133b deregulation is associated with overall survival and metastasis in colorectal cancer. Int J Oncol.

[85] Karatas OF, Guzel E, Suer I, Ekici ID, Caskurlu T, Creighton CJ, Ittmann M, Ozen M (2014). miR-1 and miR-133b are differentially expressed in patients with recurrent prostate cancer. PLoS One.

[86] Chan M, Liaw CS, Ji SM, Tan HH, Wong CY, Thike AA, Tan PH, Ho GH, Lee AS (2013). Identification of circulating microRNA signatures for breast cancer detection. Clin Cancer Res.

[87] Chen S, Zhu J, Yu F, Tian Y, Ma S, Liu X (2015). Combination of miRNA and RNA functions as potential biomarkers for gastric cancer. Tumour Biol.

[88] Zhang C, Yao C, Li H, Wang G, He X (2014). Serum levels of microRNA-133b and microRNA-206 expression predict prognosis in patients with osteosarcoma. Int J Clin Exp Pathol.

[89] Guzel E, Karatas OF, Semercioz A, Ekici S, Aykan S, Yentur S, Creighton CJ, Ittmann M, Ozen M (2015). Identification of microRNAs differentially expressed in prostatic secretions of patients with prostate cancer. Int J Cancer.

[90] Wu Y, Crawford M, Yu B, Mao Y, Nana-Sinkam SP, Lee LJ (2011). MicroRNA delivery by cationic lipoplexes for lung cancer therapy. Mol Pharm.

[91] Wu J, Yang T, Li X, Yang Q, Liu R, Huang J, Li Y, Yang C, Jiang Y. (2013). Alteration of serum miR-206 and miR-133b is associated with lung carcinogenesis induced by 4-(methylnitrosamino)-1-(3-pyridyl)-1-butanone. Toxicol Appl Pharmacol.

[92] Stumpfova Z, Hezova R, Meli AC, Slaby O, Michalek J (2014). MicroRNA profiling of activated and tolerogenic human dendritic cells. Mediators Inflamm.

[93] Navon R, Wang H, Steinfeld I, Tsalenko A, Ben-Dor A, Yakhini Z (2009). Novel rank-based statistical methods reveal microRNAs with differential expression in multiple cancer types. PLoS One.

[94] Bandres E, Cubedo E, Agirre X, Malumbres R, Zarate R, Ramirez N, Abajo A, Navarro A, Moreno I, Monzo M, Garcia-Foncillas J (2006). Identification by Real-time PCR of 13 mature microRNAs differentially expressed in colorectal cancer and non-tumoral tissues. Mol Cancer.

[95] Kara M, Yumrutas O, Ozcan O, Celik OI, Bozgeyik E, Bozgeyik I, Tasdemir S (2015). Differential expressions of cancer-associated genes and their regulatory miRNAs in colorectal carcinoma. Gene.

[96] Wong TS, Liu XB, Wong BY, Ng RW, Yuen AP, Wei WI (2008). Mature miR-184 as Potential Oncogenic microRNA of Squamous Cell Carcinoma of Tongue. Clin Cancer Res.

[97] Wei Y, He R, Wu Y, Gan B, Wu P, Qiu X, Lan A, Chen G, Wang Q, Lin X, Chen Y, Mo Z (2016). Comprehensive investigation of aberrant microRNA profiling in bladder cancer tissues. Tumour Biol.

[98] Pignot G, Cizeron-Clairac G, Vacher S, Susini A, Tozlu S, Vieillefond A, Zerbib M, Lidereau R, Debre B, Amsellem-Ouazana D, Bieche I (2013). microRNA expression profile in a large series of bladder tumors: identification of a 3-miRNA signature associated with aggressiveness of muscle-invasive bladder cancer. Int J Cancer.

[99] Ichimi T, Enokida H, Okuno Y, Kunimoto R, Chiyomaru T, Kawamoto K, Kawahara K, Toki K, Kawakami K, Nishiyama K, Tsujimoto G, Nakagawa M, Seki N (2009). Identification of novel microRNA targets based on microRNA signatures in bladder cancer. Int J Cancer.

[100] Song T, Xia W, Shao N, Zhang X, Wang C, Wu Y, Dong J, Cai W, Li H (2010). Differential miRNA expression profiles in bladder urothelial carcinomas. Asian Pac J Cancer Prev.

[101] Savarimuthu Francis SM, Davidson MR, Tan ME, Wright CM, Clarke BE, Duhig EE, Bowman RV, Hayward NK, Fong KM, Yang IA (2014). MicroRNA-34c is associated with emphysema severity and modulates SERPINE1 expression. BMC Genomics.

[102] Chen Z, Saad R, Jia P, Peng D, Zhu S, Washington MK, Zhao Z, Xu Z, El-Rifai W (2013). Gastric adenocarcinoma has a unique microRNA signature not present in esophageal adenocarcinoma. Cancer.

[103] Saad R, Chen Z, Zhu S, Jia P, Zhao Z, Washington MK, Belkhiri A, El-Rifai W (2013). Deciphering the unique microRNA signature in human esophageal adenocarcinoma. PLoS One.

[104] Liu X, Chen Z, Yu J, Xia J, Zhou X (2009). MicroRNA profiling and head and neck cancer. Comp Funct Genomics.

[105] Namlos HM, Meza-Zepeda LA, Baroy T, Ostensen IH, Kresse SH, Kuijjer ML, Serra M, Burger H, Cleton-Jansen AM, Myklebost O (2012). Modulation of the osteosarcoma expression phenotype by microRNAs. PLoS One.

[106] Missiaglia E, Shepherd CJ, Patel S, Thway K, Pierron G, Pritchard-Jones K, Renard M, Sciot R, Rao P, Oberlin O, Delattre O, Shipley J (2010). MicroRNA-206 expression levels correlate with clinical behaviour of rhabdomyosarcomas. Br J Cancer.

[107] Fu HL, Wu DP, Wang XF, Wang JG, Jiao F, Song LL, Xie H, Wen XY, Shan HS, Du YX, Zhao YP (2013). Altered miRNA expression is associated with differentiation, invasion, and metastasis of esophageal squamous cell carcinoma (ESCC) in patients from Huaian, China. Cell Biochem Biophys.

[108] Kowalewska M, Bakula-Zalewska E, Chechlinska M, Goryca K, Nasierowska-Guttmejer A, Danska-Bidzinska A, Bidzinski M (2013). microRNAs in uterine sarcomas and mixed epithelial-mesenchymal uterine tumors: a preliminary report. Tumour Biol.

[109] Hidaka H, Seki N, Yoshino H, Yamasaki T, Yamada Y, Nohata N, Fuse M, Nakagawa M, Enokida H (2012). Tumor suppressive microRNA-1285 regulates novel molecular targets: aberrant expression and functional significance in renal cell carcinoma. Oncotarget.

[110] Saito K, Inagaki K, Kamimoto T, Ito Y, Sugita T, Nakajo S, Hirasawa A, Iwamaru A, Ishikura T, Hanaoka H, Okubo K, Onozaki T, Zama T (2013). MicroRNA-196a is a putative diagnostic biomarker and therapeutic target for laryngeal cancer. PLoS One.

[111] Servin-Gonzalez LS, Granados-Lopez AJ, Lopez JA (2015). Families of microRNAs Expressed in Clusters Regulate Cell Signaling in Cervical Cancer. Int J Mol Sci.

[112] de Cubas AA, Leandro-Garcia LJ, Schiavi F, Mancikova V, Comino-Mendez I, Inglada-Perez L, Perez-Martinez M, Ibarz N, Ximenez-Embun P, Lopez-Jimenez E, Maliszewska A, Leton R, Gomez Grana A (2013). Integrative analysis of miRNA and mRNA expression profiles in pheochromocytoma and paraganglioma identifies genotype-specific markers and potentially regulated pathways. Endocr Relat Cancer.

[113] Ellermeier C, Vang S, Cleveland K, Durand W, Resnick MB, Brodsky AS (2014). Prognostic microRNA expression signature from examination of colorectal primary and metastatic tumors. Anticancer Res.

[114] Iorio MV, Croce CM (2012). MicroRNA dysregulation in cancer: diagnostics, monitoring and therapeutics. A comprehensive review. EMBO Mol Med.

[115] Lv LV, Zhou J, Lin C, Hu G, Yi LU, Du J, Gao K, Li X (2015). DNA methylation is involved in the aberrant expression of miR-133b in colorectal cancer cells. Oncol Lett.

[116] Niederwieser C, Kohlschmidt J, Volinia S, Whitman SP, Metzeler KH, Eisfeld AK, Maharry K, Yan P, Frankhouser D, Becker H, Schwind S, Carroll AJ, Nicolet D (2015). Prognostic and biologic significance of DNMT3B expression in older patients with cytogenetically normal primary acute myeloid leukemia. Leukemia.

[117] Ribas J, Ni X, Haffner M, Wentzel EA, Salmasi AH, Chowdhury WH, Kudrolli TA, Yegnasubramanian S, Luo J, Rodriguez R, Mendell JT, Lupold SE (2009). miR-21: an androgen receptor-regulated microRNA that promotes hormone-dependent and hormone-independent prostate cancer growth. Cancer Res.

[118] Yang S, Zhang J, Zhang Y, Wan X, Zhang C, Huang X, Huang W, Pu H, Pei C, Wu H, Huang Y, Huang S, Li Y (2015). KDM1A triggers androgen-induced miRNA transcription via H3K4me2 demethylation and DNA oxidation. Prostate.

[119] Lin CW, Li XR, Zhang Y, Hu G, Guo YH, Zhou JY, Du J, Lv L, Gao K, Zhang Y, Deng H (2014). TAp63 suppress metastasis via miR-133b in colon cancer cells. Br J Cancer.

[120] Huang TC, Renuse S, Pinto S, Kumar P, Yang Y, Chaerkady R, Godsey B, Mendell JT, Halushka MK, Civin CI, Marchionni L, Pandey A (2015). Identification of miR-145 targets through an integrated omics analysis. Mol Biosyst.

[121] Quinn JJ, Chang HY (2016). Unique features of long non-coding RNA biogenesis and function. Nat Rev Genet.

[122] Cesana M, Cacchiarelli D, Legnini I, Santini T, Sthandier O, Chinappi M, Tramontano A, Bozzoni I (2011). A long noncoding RNA controls muscle differentiation by functioning as a competing endogenous RNA. Cell.

[123] Coarfa C, Fiskus W, Eedunuri VK, Rajapakshe K, Foley C, Chew SA, Shah SS, Geng C, Shou J, Mohamed JS, O'Malley BW, Mitsiades N (2016). Comprehensive proteomic profiling identifies the androgen receptor axis and other signaling pathways as targets of microRNAs suppressed in metastatic prostate cancer. Oncogene.

