# Supplementary material for: miR-133b, a particular member of myomiRs, coming into playing its unique pathological role in human cancer
Source: Oncotarget. 2017 Mar 31;8(30):50193–208. doi: 10.18632/oncotarget.16745 (PMC5564843; doi:10.18632/oncotarget.16745)
Supplement: Supplementary file 1 [file oncotarget-08-50193-s001.pdf]

miR-133b, a particular member of myomiRs, coming into playing its unique pathological role in human cancer

Supplementary Materials

Supplementary Table 1: The target genes of miR-133b. Sheet 1:the target genes which processed from five miRNA prediction software programs (TargetScan, PicTar, PITA, miRanda and RNA22) and supported by CLIP-Seq data,more details can be found in starBase v2.0. **Sheet 2** and **Sheet 3** provided the experimentally validated target genes from the DIANA-TarBase v7.0 and miRTarBase 6.0. **Sheet 4** provided the intersection of starBase v2.0, DIANA-TarBase v7.0 and miRTarBase 6.0. See Supplementary_Table_1

Supplementary Table 2: The enrichment analysisoftarget genes of miR-133b. Sheet 1: KEGG pathway enrichment analysis; **Sheet 2:** OMIM_Disease enrichment analysis.All result proceed from Enrichr. See Supplementary_Table_2
